# Leptin, resistin and visfatin as useful predictors of gestational diabetes mellitus

**DOI:** 10.1186/s12944-019-1169-2

**Published:** 2019-12-13

**Authors:** Ahmed Tijani Bawah, Mohammed Mustapha Seini, Albert Abaka-Yawason, Huseini Alidu, Salifu Nanga

**Affiliations:** 1 Department of Medical Laboratory Science, School of Allied Health Sciences, University of Health and Allied Health Sciences, PMB 31, Ho, Ghana; 2 Laboratory Department, Greater Accra Regional Hospital, Accra, Ghana; 3 School of Basic and Biomedical Science, University of Health and Allied Science, Ho, Ghana

**Keywords:** Adipokines, Pregnancy, Glucose intolerance

## Abstract

**Background:**

Lipids and adipokines including leptin, resistin and visfatin play various roles in the pathophysiology of Gestational Diabetes Mellitus (GDM). This study was aimed at determining whether serum leptin, resistin and visfatin are significantly altered during the first trimester of pregnancies that subsequently develop GDM and whether such changes are useful in predicting the disease.

**Methods:**

This was a case-case control study which compared first trimester biochemical and anthropometric parameters in 70 pregnant women who subsequently developed GDM and 70 pregnant women without GDM at the Volta Regional Hospital, Ho, Ghana. Lipid profile and some selected adipokines were analyzed and first trimester body mass index (BMI) was determined.

**Results:**

There were significant differences (*p* < 0.05) in leptin, resistin, and visfatin as well as significant dyslipidemia among those with GDM compared to those without GDM. Furthermore, the area under the Receiver Operating Characteristic Curves (AUCs) for leptin, resistin and visfatin were; 0.812, 0.836 and 0.799 respectively. Increased first trimester leptin (OR = 1.166; CI = 1.104–1.233; *p < 0.0001*), resistin (*p < 0.0001*) and visfatin (*p < 0.0001*) were associated with GDM.

**Conclusion:**

Hyperleptinemia, hyperesistinemia and hypervisfatinemia precede GDM and can serve as good predictive indices for gestational diabetes mellitus.

## Background

The main objective of the current study was to explore the association and accuracy of some adipokines in predicting GDM. GDM is the occurrence of glucose intolerance which is diagnosed for the first time during pregnancy [1]. Available data point to the involvement of some adipocytokines in glucose metabolism [2]. Adipokines are made up of peptides and proteins with diverse functions and include mediators of vascular hemostasis [3], blood pressure regulation and angiogenesis [4]. They also include cytokines [5, 6], chemokines like monocyte chemoattractant protein-1 [7] and hormones such as resistin [8] and leptin [9, 10] which are all involved in glucose homeostasis.

Hyperleptinemia in early stages of pregnancy may be predictive of an increased risk of developing GDM in the later stages of pregnancy irrespective of obese status [11].

Leptin levels in amniotic fluid at 15–17 weeks of gestation is significantly higher in women with GDM as compared to women with euglycemia throughout the period of pregnancy [12]. Other authors have reported different findings about leptin in GDM; Simmons and Breier [13] reported no difference in serum concentration of leptin between normal pregnancies and pregnancies complicated by GDM while some researchers reported decreased plasma leptin in women with GDM [14].

Available information on plasma concentrations of resistin in gestational diabetes mellitus have not been consistent; some reports indicated no alteration of resistin concentration in GDM as compared to healthy pregnant women [15], while others have reported increased [16] or decreased [17] levels in women with GDM.

Visfatin displays nicotinamide phosphoribosyltransferase activity [18] and is secreted from the epithelial cells of amniotic sac during pregnancy [19]. Visfatin levels during pregnancy complicated by glucose intolerance may be increased [20] or decreased [21] and also involved in apoptosis [19]. The main aim of this study was to determine whether in the first trimester of pregnancy, serum concentrations of leptin, resistin and visfatin are altered in pregnancies that subsequently develop GDM and whether such changes are significant to the extent of elucidating pregnancies that are likely to develop GDM.

Several risk factors have been associated with GDM including; earlier diagnosis of GDM, advanced maternal age and history of type 2 diabetes mellitus among first degree relatives of the pregnant woman [22]. Others are; ethnic background, overweight and obesity, as well as macrosomia from previous pregnancies [23].

Lipid profile changes in normal pregnancies are characterized by hypercholesterolemia and hypertriglyceridemia and elevated levels of very low density lipoprotein-cholesterol (VLDL-C) in the liver as a result of increased level of estrogen [24]. The reduction in lipoprotein lipase (LPL) activity due to down regulation of the LPL gene expression caused by estrogen during pregnancy decreases the clearance of VLDL-C [25]. GDM affects the cardiovascular and metabolic processes of the baby and in-utero hyperinsulinemia independently predicts glucose intolerance in childhood [26]. The changes in the lipid profile and the disparities in the metabolism of some adipokines as reported by various studies requires close scrutiny of their roles in the pathophysiology of GDM. Therefore, this study sought to determine whether in the first trimester of pregnancy, metabolism of leptin, resistin, visfatin and lipids are affected in pregnancies that subsequently develop GDM and whether these variations can be used as basis to predict GDM so as to elicit interventions early enough to save the mother and the baby.

## Materials and methods

### Study design and site

This prospective case-control study was carried out from August 2014 to August 2016 at the antenatal clinic of the Volta Regional Hospital in the Ho Municipality of Ghana.

### Study population

Pregnant women who visited the hospital between 11 and 13 weeks of gestation for their first routine antenatal care and agreed to take part in this study were recruited. In this study, we recorded maternal characteristics and also took blood samples of all participants.

A standard pretested questionnaire was used to obtain information on demographic data, family history of diabetes mellitus, parity, gravidity, history of stillbirth and miscarriages.

#### Inclusion criteria

Women who developed GDM with no preexisting glucose intolerance.

#### Exclusion criteria

Women with liver disease, kidney disease and chronic diseases such as diabetes mellitus and hypertension.

### Anthropometric measurement

Height was measured without participants wearing foot wear using a stadiometer to the nearest 0.5 cm with the study participants standing upright and heels put together and the head in the horizontal plane and weight was measured in kilograms with participants wearing light clothing using the bioimpedance analyzer (BIA); (BSD01, Pure Pleasure, a division of the Stingray Group, Capetown, South Africa). The BMI, was determined using the BIA according to the manufacturer’s instruction. All measurements and samples were taken between 7:00 am and 8:00 am after an overnight fast for a period of 10 to 16 h [27].

### Biochemical analysis

Five milliliters of venous blood samples were taken from all participants between 11 and 13 weeks of gestation, after an overnight fast and 2 ml put into potassium-EDTA anti-coagulated tube and the remaining 3 ml into serum separator tubes. The blood samples were then centrifuged to obtain serum samples which were stored in several aliquots at − 80 °C until sample analysis. Hemoglobin A1c was determined in all participants and those who had values ≥6.5% were deemed to have pre-existing diabetes mellitus and therefore excluded from the study. Lipid profile and the plasma glucose were determined using the Vitros dry chemistry analyzer (Ortho- Clinical Diagnostics, Johnson & Johnson, High Wycombe, UK). Leptin, Resistin and Visfatin in the participants were estimated quantitatively by sandwich Enzyme-linked Immunosorbent Assay technique (Elabscience Biotechnology Co. Ltd., Wu Han, People’s Republic of China). Repeated freezing and thawing of samples were avoided as much as possible.

### Outcome variable

The primary outcome was GDM, defined as the occurrence of hyperglycemia in pregnant women who have never had diabetes. We conducted a 2-h, 75-g Glucose challenge test (GCT) on all participants at 26 ± 2.6 weeks gestation. If the GCT value was ≥7.8 mmol/l, a 3-h, 100-g oral glucose tolerance test (OGTT) was conducted. Diagnosis of GDM was based on the criteria of the American Diabetes Association [28].

### Power and statistical analysis

All data analyses were performed using the SPSS software (version 11.0 systat, Inc. Germany) and GraphPad Prism, (version 5.0, San Diego California, USA). Data was presented as mean ± SD. Area under receiver operating characteristic curves (AUCs) were determined for each biomarker. Logistic regression was used to evaluate the effect of maternal characteristics on the predictive abilities of the adipokines. In all the statistical analysis, a value of *p* < 0.05 was considered to be significant and at a 95% confidence interval.

The area under the receiver operating characteristic curve (AUC) is usually regarded as a measure of the accuracy of a test/marker. Therefore, if the AUC is 50% or less, then the result can be seen as a random guessing and therefore not significant. This is represented by diagonal line in the ROC plot [29]. We tested leptin, resistin and visfatin to see whether each of them had some accuracy (AUC ~ 60%). Therefore, we determined minimum power of 80% with alpha 5%.

Pregnant women in this hospital are routinely screened for GDM between 24 and 28 weeks of gestation for GDM. We had earlier reported a GDM prevalence of 7% in our previous study in the Volta Region of Ghana [30]; therefore, we assumed that 7% of the study population would develop GDM. Consequently, out of a total of 1047 participants 70 developed GDM. We therefore took 70 cases and 70 randomly selected controls making a total of 140 pregnant participants. The sample size and power calculation were performed using SAS® %ROCPOWER macro [31].

## Results

The baseline anthropometrics, lipids and adipokines as stratified by GDM (Table 1) indicated that the mean age and BMI of those who developed GDM were significantly higher than those without GDM (*p = 0.023*) and *(p = 0.004*) respectively. The first trimester lipid profile also showed significant differences (*p < 0.05*) in the triglycerides (TG), total cholesterol (TC), low density lipoprotein cholesterol (LDL-C) and very low density lipoprotein cholesterol (VLDL-C). There was, however, no significant difference in the high density lipoprotein cholesterol (*p = 0.319*) between the GDM group and those without GDM. Assessment of adipokine status during the first trimester also showed significantly higher leptin, resistin and visfatin among those who subsequently developed GDM as compared to those without GDM (*p < 0.0001*).

**Table 1:** Comparison of anthropometric and biochemical markers among the participants

Variable	GDM (*N* = 70)	NO GDM (N = 70)	*P* values
AGE (Years)	30.8714 ± 5.743	28.7571 ± 5.094	0.023
BMI (Kg/m^2^)	27.5257 ± 3.931	25.5843 ± 3.843	0.004
TG (mmol/L)	2.7346 ± 0.706	1.5123 ± 0.686	< 0.0001
TC (mmol/L)	7.2029 ± 1.204	5.1801 ± 1.556	< 0.0001
HDL-C (mmol/L)	1.3239 ± 0.958	1.4816 ± 0.908	0.319
LDL-C (mmol/L)	4.6933 ± 1.436	3.269 ± 1.574	< 0.0001
VLDL-C (mmol/L)	1.2421 ± 0.320	0.6899 ± 0.316	< 0.0001
Leptin (ng/ml)	35.0434 ± 8.700	21.9352 ± 9.192	< 0.0001
Resistin (ng/ml)	9.7129 ± 2.314	6.4049 ± 2.441	< 0.0001
Visfatin (ng/ml)	8.7714 ± 4.010	4.6725 ± 2.739	< 0.0001

*BMI* Body mass index, *TG* Triglycerides, *TC* Total cholesterol, *HDL-C* High density lipoprotein cholesterol, LDL-C Low density lipoprotein cholesterol, VLDL-C cholesterol. Data is presented as mean ± SD

Correlation of anthropometric and biochemical parameters of participants are shown in (Table 2). With the exception of HDL which had negative correlation with GDM, all the parameters in the lipid panel had significantly positive correlations with GDM. Age correlated positively with BMI (r = 0.348, *p < 0.0001*), GDM (r = 0.192, *p = 0.023*), leptin (r = 0.221, *p = 0.009*) and visfatin (r = 0.191, *p* = 0.024). A similar correlation was observed between BMI and GDM (r = 0.244, *p = 0.004*). However, these correlations were largely weak (Table 2). There were significant positive correlations between leptin and GDM, resistin and GDM as well as between visfatin and GDM.

**Table 2:** Correlation of demographic, clinical, and biochemical parameters of participants

		AGE	BMI	TG	TCHOL	HDL	LDL	VLDL	LP	RESTN	VISF	GDM
AGE	R	1	0.348^a^	0.019	0.190^b^	−.288^a^	0.202^b^	0.153	0.221^a^	0.071	.191^b^	0.192^b^
	P value		0	0.738	0.024	0.001	0.017	0.071	0.009	0.402	0.024	0.023
BMI	R		1	0.130^b^	0.069	−0.112	0.083	0.091	0.159	0.136	0.128	0.244^a^
	P value			0.021	0.42	0.186	0.332	0.285	0.06	0.108	0.132	0.004
TG	R			1	0.502^a^	−0.107	0.214^a^	.957^a^	0.120^b^	0.115^b^	.159^a^	0.325^a^
	P value				0	0.059	0	0	0.035	0.043	0.005	0
TCHOL	R				1	−.421^a^	0.859^a^	.664^a^	0.320^a^	0.325^a^	0.219^a^	0.667^a^
	P value					0	0	0	0	0	0.009	0
HDL	R					1	−.440^a^	−.366^a^	−.229^a^	−.257^a^	−.287^a^	−0.334^a^
	P value						0	0	0.007	0.002	0.001	0
LDL	R						1	0.383^a^	.310^a^	.399^a^	0.276^a^	0.581^a^
	P value							0	0	0	0.001	0
VLDL	R							1	0.394^a^	0.276^a^	0.305^a^	0.603^a^
	P value								0	0.001	0	0
LP	R								1	0.608^a^	.719^a^	0.594^a^
	P value									0	0	0
RESTN	R									1	0.562^a^	0.574^a^
	P value										0	0
VISF	R										1	0.515^a^
	P value											0
GDM	R											1
	P value											

*TG* Triglycerides, *TCHOL* Total cholesterol, *HDL* High density lipoprotein cholesterol, *LDL* Low density lipoprotein cholesterol, *VLDL* Very low density lipoprotein cholesterol, *ADP* Adiponectin, *LP* Leptin, *RESTN* Resistin, *VISF* Visfatin, *GDM* Gestational diabetes mellitus

^a^Correlation is significant at the 0.01 level (2-tailed)

^b^Correlation is significant at the 0.05 level (2-tailed)

**Fig. 1:**
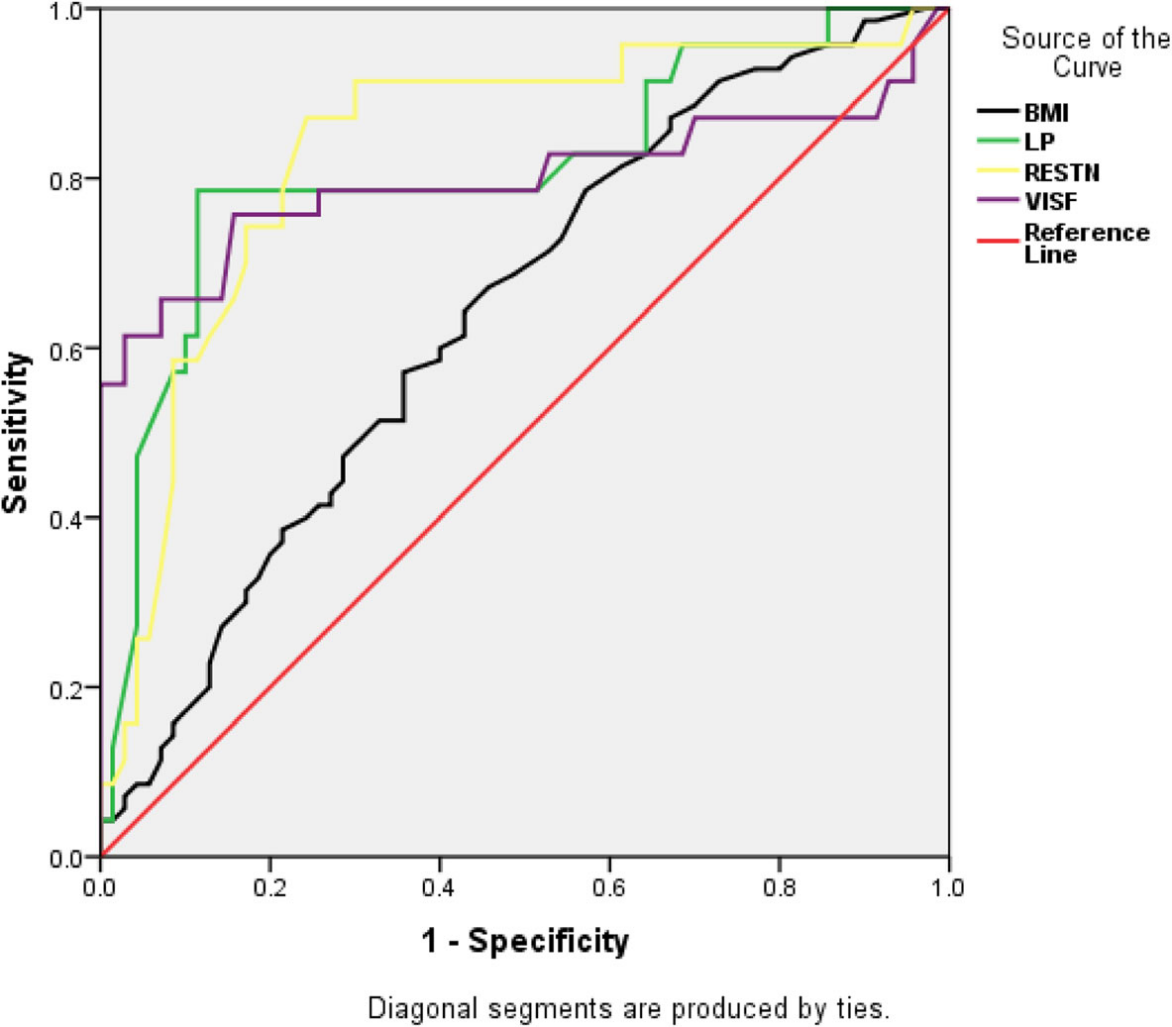
ROC curves for Body mass index (BMI), Leptin (LP), Resistin (RESTN) and Visfatin (VISF)

The area under the receiver operator characteristic curve as shown in Table 3 and Fig. 1 indicates the ability of the adipokines to positively predict GDM. The areas under the curve for leptin resistin and visfatin are; 0.812, 0.836 and 0.799 respectively. The sensitivity, specificity and threshold levels of these adipokines are also shown in Table 3. Analyses of the results indicate that at a cutoff point of ≥18.9 ng/ml, leptin showed a sensitivity and specificity of 95.7 and 68.6% respectively in the prediction of GDM. Similarly, resistin and visfatin showed high sensitivity and specificity in predicting GDM at threshold level of ≥5.3 ng/ml and ≥ 2.8 ng/ml respectively However, BMI showed a sensitivity and specificity of 51.4 and 67.1% respectively at a cut of point of ≥27 kg/m^2^ (Table 3).

**Table 3:** Area under ROC (AUC), threshold level, sensitivity and specificity of the biochemical markers in GDM

	AUC	Cutoff point	Sensitivity	Specificity
Leptin (ng/ml)	0.812	≥ 18.90	95.7	68.6
Resistin (ng/ml)	0.836	≥ 5.30	95.7	61.4
Visfatin (ng/ml)	0.799	≥ 2.80	87.1	70
BMI (Kg/m^2^)	0.642	≥ 27.05	51.43	67.14

Multivariate logistic regression of factors associated with GDM is shown in Table 4. High BMI (OR = 1.128; CI = 1.012–1.257, *P = 0.03*), previous miscarriages (OR = 3.143; CI = 1.22–8.095; *P = 0.018*) history of stillbirth (OR = 4.542; CI = 1.159–17.795; *P = 0.03*) and previous caesarean operation (OR = 13.716; CI = 4.146–45.382; *P < 0.0001*) were significantly associated with the development of GDM. However, parity (*P = 0.494*) and gravidity (*P = 0.477*) were not significantly associated with GDM among our participants. Elevated serum leptin (OR = 1.166; CI = 1.104–1.233; *p < 0.0001*), resistin (OR = 1.772; CI = 1.432–2.192; *p < 0.0001*) and visfatin (OR = 1.342; CI = 1.185–1.518; *p < 0.0001*) were associated with GDM. However, first trimester TG (*p = 0.543*) and TC (*p = 0.588*) were not associated with the development GDM.

**Table 4:** Multivariate logistic regression of factors and biochemical markers associated with GDM

Variable	Regression coefficient (β)	OR (95% CI)	*P value*
AGE	0.043	1.044 (0.964–1.13)	0.292
BMI	0.12	1.128 (1.012–1.257)	0.03
RWD	−0.611	0.543 (0.18–1.638)	0.278
Parity	−0.319	0.727 (0.292–1.812)	0.494
MC	1.145	3.143 (1.22–8.095)	0.018
SB	1.513	4.542 (1.159–17.795)	0.03
CS	2.619	13.716 (4.146–45.382)	< 0.0001
Gravidity	−0.304	0.738 (0.319–1.705)	0.477
Leptin	0.154	1.166 (1.104–1.233)	< 0.0001
Resistin	0.572	1.772 (1.432–2.192)	< 0.0001
Visfatin	0.294	1.342 (1.185–1.518)	< 0.0001
TG	14.42	183 (0.00–2.750)	0.543
TC	0.282	1.325 (0.478–3.677)	0.588

*BMI* Body mass index, *RWD* Relatives with diabetes, *MC* Miscarriages, *CS* caesarean operation, *SB* Stillbirth, *TG* Triglycerides, *TC* Total cholesterol

## Discussion

We assessed the association between first trimester leptin, resistin, visfatin, BMI and maternal characteristics and the development of gestational diabetes mellitus in the Ho municipality. Our report indicated that leptin, resistin and visfatin were all independently elevated in women with GDM many weeks before the diagnosis of the disease. We also report that the elevations in these adipokines were independent of maternal age, family history of diabetes, parity and lipid levels. These results showed that serum leptin, resistin, and visfatin were higher among those who subsequently developed gestational diabetes mellitus. Serum lipids (TG, TC, LDL, and VLDL) were higher among those who developed GDM and high BMI, previous miscarriages, stillbirth as well as previous cesarean operation were independently associated with GDM.

Increased obesity had been reported previously to increase the risk of developing GDM by about 3 fold in pregnant women [22] and this is in agreement with this present study which alludes to the fact that participants with GDM were more obese than the healthy controls (Table 1) but not in consonant with a study carried out in Korle-Bu, Accra, which reported no significant difference in the BMI of women with GDM and those without GDM [32]. In an earlier study we demonstrated that higher maternal pre-pregnancyBMI was linked to the development of preeclampsia, which usually occurs during the second trimester [33]. It is therefore not surprising that this study also provides additional evidence of the link between maternal BMI and pregnancy complications like GDM which develops after 24 weeks of gestation.

Our study revealed strong positive correlations between lipids and GDM with the exception of HDL which showed negative correlation. This is in consonance with previous reports which reported strong correlations between lipids and glucose intolerance during pregnancy [34, 35]. Hyperlipidemia could contribute to the insulin resistance which is a feature of gestational diabetes mellitus; and so when there is high plasma lipids especially hypertriglyceridemia, the consequence could be glucose intolerance leading to GDM. Our study also showed that significantly higher serum leptin determined between 11 and 13 weeks of gestation existed in women who later developed GDM than the healthy controls and could be used to predict the occurrence of GDM (Table 1 and Table 3). This study also reports high sensitivity, specificity and high accuracy (Table 3) of using first trimester leptin measurement in the prediction of GDM. The Normal physiological functions of leptin are; regulation of inflammation, immune response, endocrine function, reproduction and angiogenesis [36]. Leptin modulates pancreatic β-cell function and enhances peripheral insulin sensitivity and has been established as a key regulator of glucose homeostasis, both in rodents and humans [37]. In some pregnancies, as may be the case in the participants in this study, these normal functions of leptin become disrupted. Consequently, leptin resistance develops causing insulin resistance and impaired glucose tolerance. The placenta also produces leptin during pregnancy resulting in further hyperleptinemia [38]. The hyperleptinemia due to excessive fat accumulation, fetal and placental production all contribute to leptin resistance and subsequently insulin resistance resulting in gestational diabetes mellitus in individuals who are not able to compensate for the metabolic deregulation.

Our study also revealed that significantly higher plasma resistin between 11 and 13 weeks of pregnancy existed in participants who later developed GDM as compared to their healthy control counterparts [16] but this finding is contrary to a previous report which indicated that GDM is associated with lower resistin levels [17]. The exact function of resistin in GDM is still unclear, however this finding which showed increased levels of resistin between 11 and 13 weeks of pregnancy in women who later developed GDM suggest that this adipokine may have a role to play in the pathophysiology of GDM and that hyperesistinemia possibly precede the onset/ diagnosis of GDM. It could be responsible for the initial insulin resistance that eventually result in glucose intolerance and either reduce or stabilize to levels similar to uncomplicated pregnancies after the onset of the GDM. This could be the possible reason why different researchers reported different findings.

This study has shown that during pregnancy visfatin is increased weeks before the onset of GDM and could be used as a predictive index of the disease irrespective of the maternal characteristics. This is similar to a report which found increased visfatin levels weeks before clinical diagnosis of the disease [39]. Another report indicated that visfatin levels in pregnancy complicated by glucose intolerance was significantly higher than those with normal glucose tolerance [20]. Visfatin is produced by adipose tissue, placenta and the fetal membrane [40]. The significant increase in visfatin levels in pregnancies that resulted in GDM compared to those who remained normoglycemic suggest that this peptide might be involved in the exacerbation of insulin resistance seen in normal pregnancies leading to gestational diabetes mellitus. Other reports have, however, reported lower visfatin levels [21] in GDMs compared with the controls. One study reported that visfatin levels did not vary significantly between the women with GDM and those with normal response to glucose challenge test between 26 and 33 weeks of gestation but were significantly lower in GDM than in those with normal glucose tolerance at the end of the pregnancy [41]. The finding in this study, when compared with the different results as reported by other researchers suggest that, initially visfatin may rise in pregnancy complicated by glucose intolerance but negative fee back mechanism might result in the level reducing to similar level as in the normal glucose tolerant pregnant women and may even fall further at term before probably stabilizing. More work is required to elucidate usefulness of this peptide in the prediction of GDM.

There were also significant positive correlations between leptin, resistin, and visfatin and gestational diabetes mellitus (Table 2). Advanced maternal age, obesity, family history of diabetes mellitus, history of poor obstetric outcome [23] as well as abnormal lipid levels, especially TG [42] contribute to the development of GDM. When we controlled for age and other confounders through multivariate logistic regression analysis, those who developed GDM were obese and had history of poor obstetric outcomes like miscarriages, stillbirths and caesarean operation. However, the overall abilities of these adipocytokines to positively predict GDM were not affected (Table 4).

In this study it was observed that in general, several significant changes involving first trimester triglycerides, total cholesterol, HDL cholesterol, LDL cholesterol, and VLDL cholesterol existed between cases with gestational diabetes mellitus and controls. The significant difference that existed between the lipid profiles of the GDMs and those without GDM in this study could be due to the pathophysiology of the disease. This means that changes in the lipid profile pattern precede the onset of GDM and could play a major role in the insulin resistance states of gestational diabetes mellitus. This is in agreement with a previous study in which significant differences were reported between participants with gestational diabetes mellitus and the controls [42]. It is also in consonance with studies by other researchers [43–45]. Our results are also in consonance with a previous report which indicated that TG levels are significantly raised throughout the course of pregnancy in women with GDM and that HDL levels are markedly reduced in the second and third trimesters of pregnancy [46] but their observations of small variations in TC, LDL and VLDL between women with GDM and those without GDM are not in agreement with our findings. However, one report did not find significant difference in TG between women with previous GDM cases and controls [47]. The discrepancies could be as a result of differences in method of selection of subjects for the study. The GDM group of the subjects studied by Koivunen et al., in 2001 [47], involved women with previous gestational diabetes mellitus, some of whom were treated with insulin and others with diet, and probably, the treatment as well as the time period between the time they had the GDM and the time of the study, could have affected the lipid profile patterns.

The hyperlipidemia observed could be as a result of the fact that during pregnancy, fat storage increases [48] and progesterone which increases around 20th week of gestation, act in a way to reset the lipostat in the hypothalamus leading to increase in the lipids concentration in gestational diabetes mellitus [49]. It is even possible that the increase in fat storage reported by some investigators [48] could have started much earlier in the first trimester as shown in this study where hyperlipidemia was demonstrated between 11 and 13 weeks of gestation in GDM. Therefore, these adipokines, lipids, and maternal characteristic are import factors to consider if glucose intolerance during pregnancy is to be properly controlled. The benefit of early identification of pregnant women likely to develop gestational diabetes mellitus would be to “prevent” the onset of glucose intolerance (and its inherent risks to the pregnancy) in a timely manner. This can be achieved by proposing lifestyle changes in susceptible pregnant women because lowering TG levels (by diet modification), weight reduction, and physical activity may help to prevent complications during pregnancy and adverse pregnancy outcomes [50].

## Conclusion

Leptin, resistin and visfatin are significantly increased between 11 and 13 weeks of gestation in pregnant women with glucose intolerance and these biomarkers can be used in combination with maternal characteristics for the early prediction of GDM.

## Data Availability

The datasets during and/or analyzed during the current study available from the corresponding author on reasonable request.

## Abbreviations

BMI: Body mass index
GDM: Gestational diabetes mellitus
HDL-C: High-density lipoprotein cholesterol
LDL-C: Low-density lipoprotein cholesterol
OGTT: Oral glucose tolerance test
OR: Odds ratio
SD: Standard deviation
TC: Total cholesterol
TG: Triglycerides
VLDL-C: Very low density lipoprotein cholesterol
T2DM: Type 2 diabetes mellitus
